# TOX, through a glass, darkly

**DOI:** 10.3389/fimmu.2025.1576468

**Published:** 2025-07-17

**Authors:** Patrick G. Hogan, Bruno Villalobos Reveles, Leo Josue Arteaga Vazquez

**Affiliations:** ^1^ La Jolla Institute for Immunology, La Jolla, CA, United States; ^2^ Moores Cancer Center, University of California–San Diego, La Jolla, CA, United States; ^3^ Program in Immunology, University of California–San Diego, La Jolla, CA, United States

**Keywords:** TOX, transcription factor, mechanism, HMG domain, HMG-box protein, T cell, CD8+ T cell, exhaustion

## Abstract

The transcription factor TOX has attracted attention in recent years for its role in CD8^+^ T cell exhaustion. In fact, TOX was known historically for its diverse roles in immune cell biology. Here, we inquire into the basis for this versatility, and propose that one main consideration is that TOX is an HMG-box transcription factor. We discuss some mechanisms that other HMG-box transcription factors employ to perform their cellular functions, as examples of the range of mechanisms TOX might employ in furthering T cell exhaustion. This inquiry begins with the literature placing TOX as a central player in CD8^+^ T cell exhaustion and in other immune cell processes. An understanding of TOX as a transcription factor has to be organized around its binding to relevant target sites in DNA. Thus, we next cover the reasons that TOX is classified as an HMG-box protein, the well-defined but narrow scope of what TOX shares with other HMG-box proteins, and the unequivocal evidence that binding of HMG-box proteins stabilizes kinked or bent DNA. We consider the constant features and some variables in DNA recognition by HMG-box proteins. Since binding and bending DNA is not in isolation an explanation of any biological process, we look at biological examples highlighting specific ways that HMG-box proteins drive cellular processes. Finally, we outline some lines of research that could be informative in understanding the cellular mechanisms of TOX in T cell exhaustion.

## TOX in exhausted CD8^+^ T cells

### Data that prompted attention to TOX

Converging threads of evidence made TOX a candidate regulator of T cell exhaustion. *TOX* mRNA was elevated in CD8^+^ T cells when hyporesponsiveness was induced by a constitutively active version of NFAT1 (1), in CD8^+^ tumor-infiltrating lymphocytes (TILs) undergoing exhaustion (2), in mouse tumor antigen-specific CD8^+^ TILs or CD8^+^ CAR TILs (3, 4), and in exhausted TILs recovered from human melanomas (5). *TOX* mRNA was similarly elevated when exhaustion was caused by chronic LCMV infection (6, 7). The collected evidence was summarized in (8, Supplementary Figure S1). The mRNA findings were backed up by the progressive elevation of TOX protein as exhaustion proceeded in TILs infiltrating mouse B16 melanomas or tamoxifen-inducible liver cancers (8, 9), and by the expression of TOX protein in CD8^+^ TILs from human tumors (8–10). There was high sustained expression of TOX protein in LCMV-specific T cells from chronic viral infections (10).

### TOX is involved in implementing T cell exhaustion

Several laboratories explored the role of TOX in T cell exhaustion independently. One experimental design used CD8^+^ T cells from *TOX* ^–/–^ mice (9–12). Another design, sensitive to a concern about the effects of TOX absence on T cell development in the thymus, delivered shRNAs targeting *TOX* to CD8^+^ CAR T cells that had been isolated from *TOX2* ^–/–^ mice (8). TOX^–/–^ TILs exhibited very little upregulation of inhibitory receptors, but were nevertheless poorly functional, as assessed by IFNγ and TNF expression and by target cell lysis (9). Tumor control was not examined in these experiments, because the tumor model involved the induction of widespread liver cancer. CAR TILs prepared with sh*TOX* in a *TOX2* ^–/–^ background showed reduced expression of inhibitory receptors, along with better preservation of the ability to make IFNγ and TNF and to lyse target cells (8). These CAR T cells were also more effective in controlling a tumor than control CAR T cells, completely clearing the tumor in many cases. There were characteristic changes in TIL gene expression in the absence of TOX, with a few notable differences between the two datasets that might reflect contributions from TOX2 in one case and contributions from residual TOX in the other (8, 9). Gene expression in LCMV-specific P14 T cells early in a chronic infection with LCMV clone 13 was distinguished by a strong effector-like signature (10).

A consistent observation in both tumors and chronic infection was that complete loss of TOX resulted in failure to maintain a responding T cell population beyond 1–3 weeks after T cell adoptive transfer (9–11). Thus TOX is crucial to the long term survival of exhausted CD8^+^ T cells. This has been traced to a pathology of progenitor-exhausted T cells in the absence of TOX (11, 13). In contrast, TOX is dispensable for the initial activation and expansion of CD8^+^ effector T cells and differentiation of memory T cells in the primary response to acute infections (9, 10).

### Level of TOX expression

Haploinsufficient P14 CD8^+^ T cells— presumably with about half the normal level of TOX protein— controlled B16-gp33 tumor growth better than wildtype P14 cells (10), and bulk RNA-seq data showed that there was residual *Tox* mRNA on day 8 after transfer in the sh*TOX*-treated CAR TILs that were effective in controlling tumor growth (8). It would be of major biological and practical importance if there were different threshold levels of TOX for TOX-dependent T cell survival and TOX-dependent T cell hyporesponsiveness, but current evidence is not determinative on this point. Signaling interventions that modulate the *TOX* locus opening characteristic of exhausted CD8^+^ TILs (1–3, 9) might offer a path forward. For example, BATF overexpression in TILs attenuates *TOX* locus opening and TOX expression (14), and CD4^+^ T cell help in CD8^+^ T cell–dendritic cell–CD4^+^ T cell triads within a tumor prevents *TOX* locus opening and ameliorates CD8^+^ T cell exhaustion (15). It has not been determined yet to what extent reduced TOX expression is causative for the lower expression of inhibitory receptors and improved effector function, but these cases might provide an avenue to test for a causal link.

### Unanswered questions

Defining the contributions of TOX as a transcription factor in T cell exhaustion requires connecting TOX to individual target genes. A strong case can be made that the *Pdcd1* locus is a direct transcriptional target of TOX. The exhaustion-specific -23 kb open-chromatin region upstream of the *Pdcd1* transcription start site is a functional enhancer in the EL4 cell line, a model for T cells with constitutive high expression of PD1 (16); and binding of TOX to that region has been demonstrated by ChIP-qPCR in CD8^+^ T cells ectopically expressing FLAG-tagged TOX (8). TOX might conceivably regulate other genes in the exhaustion program in a similar way. On the other hand, a plausible reading of the Alfei et al. paper (11) is that TOX is a restraint on the CD8^+^ T cell effector program in virally infected mice. The data there show that T cells lacking functional TOX have more KLRG1 expression and greater effector function during chronic LCMV infection, whereas forced expression of TOX under conditions where cells are on the borderline for developing exhaustion leads to markedly fewer cells expressing KLRG1 and an attenuated effector program. If a primary role of TOX lies in shifting the balance between effector and exhaustion programs, or in promoting the survival and expansion of specific differentiating T cell subsets, TOX might accomplish these objectives by controlling a relatively small set of direct target genes, and many genes whose expression is sensitive to TOX might not be direct targets.

## TOX in other immune cells

### TOX in memory T cells

A counterpoint to the studies of TOX and T cell exhaustion came in a report that mouse CD8^+^ memory T cells can rapidly upregulate TOX in response to either TCR stimulation or inflammatory cytokine stimulation, at the same time upregulating the effector proteins interferon-γ and granzyme B (17). Since expression of TOX was not monitored for an extended period in that report, it is not clear how the data should be related to the sustained TOX levels in exhausted T cells (8–10) or to the early transient expression of TOX at modest levels in effector T cells in acute infections (10). In the latter acute-infection experiments, effector T cells were differentiating from naïve T cells, and one simple possibility is that memory CD8^+^ T cells are more efficient than naïve CD8^+^ T cells at the rapid transient induction of TOX. TOX is also typically present in human CD8^+^ effector-memory cells (18). *Tox* mRNA levels are also elevated in tissue-resident memory CD8^+^ T cells in the small intestine (19). The observation of TOX in memory T cells is reminiscent of findings from an earlier analysis of T cell gene coexpression networks in resolving LCMV Armstrong infection and in chronic LCMV clone 13 infection (20). There, TOX expression was detected both in CD8^+^ memory T cells and in CD8^+^ exhausted T cells, but TOX was connected into different transcriptional submodules in the two cases. The three papers (17, 18, 20) articulated, at a minimum, a clear caution against conflating TOX expression with exhaustion.

### Earliest studies: TOX in thymus

**Figure 1:**
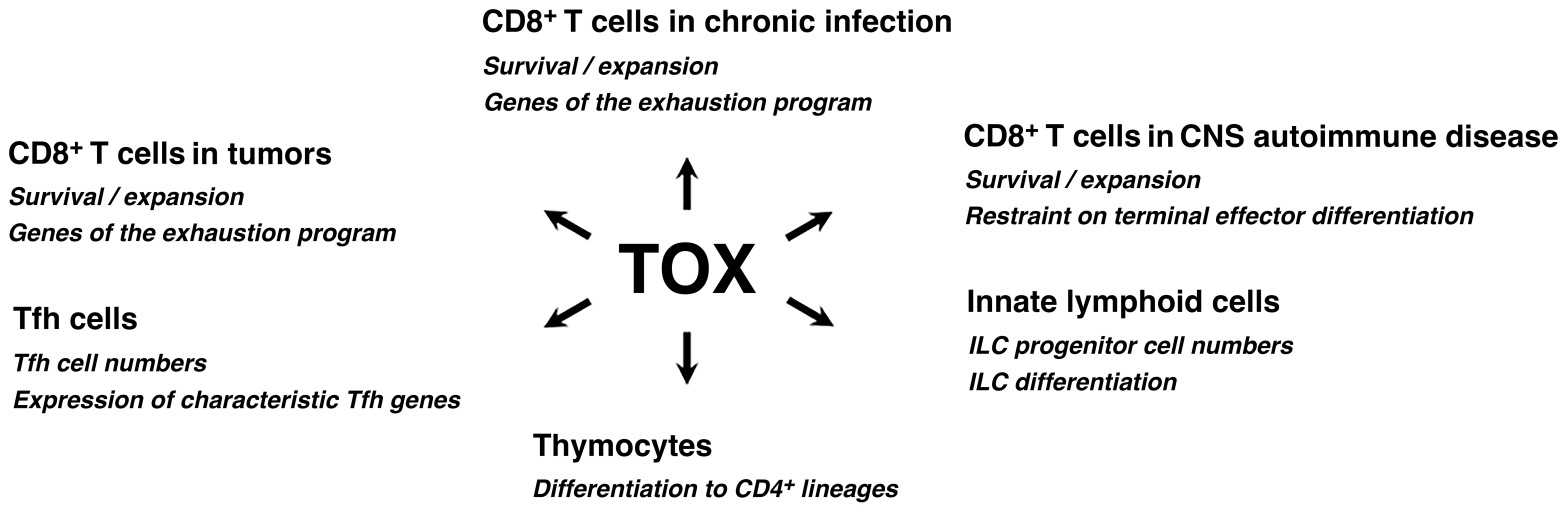
TOX acts in diverse processes in immune cells. TOX has documented roles in the development of adaptive and innate immune cells, and steers the differentiation of mature CD4^+^ and CD8^+^ T cells in specific contexts. Refer to the text for further discussion. TOX is also prominently expressed in thymocytes prior to β-selection, transiently expressed in some CD8^+^ T cells at the outset of an effector response, and upregulated in some CD8^+^ memory T cells and resident memory T cells of the small intestine (not depicted), but its biological effects in those cases have not been defined.

In fact, the caution was just a reminder of older evidence that TOX is a versatile actor in the immune system (**Figure 1**). TOX was initially discovered as a transcription factor contributing to T cell development in the thymus (21). TOX is upregulated transiently in thymocytes at the time of β-selection and again in DP thymocytes undergoing positive selection, and cells lacking functional TOX are blocked in CD4^+^ lineage development at the CD4^low^CD8^low^ > CD4^+^CD8^low^ transition (21–24).

### Other cases

The prominent role of TOX in thymocyte development is not an isolated instance. TOX is crucial to the development of lymphoid tissue inducer (LTi) cells and conventional NK cells (25), innate lymphoid cells (ILCs) (26), and follicular helper T (Tfh) cells (27). In mouse oligodendrocyte-OVA or oligodendrocyte-LCMV glycoprotein models of pathogen-triggered CNS autoimmune disease, TOX supports CD8^+^ T cell persistence in the CNS and restrains terminal effector differentiation (28, 29). In both acute and chronic viral infections, TOX is expressed early in a population of TCF1^+^TOX^+^ CD8^+^ T cells that have been characterized as multipotential progenitors (30, 31). The unequivocal message is that TOX has critical functions in diverse immune-cell contexts. Remaining questions are whether and to what extent TOX invokes common mechanisms and gene targets in carrying out these diverse functions, and, conversely, whether TOX relies on distinct mechanisms and gene targets for some individual cases.

## TOX as an HMG-box protein

### Thymocyte selection-associated high mobility group box protein

As recognized in its formal designation— thymocyte selection-associated high mobility group (HMG) box protein— TOX is grouped in the broad class of HMG box proteins. These proteins share one or more copies of an ~80-residue stretch of recognizable sequence homology with the canonical examples of the class, the proteins designated HMGB1-HMGB4 in humans. Distinct subfamilies of HMG box share have limited or no sequence homology outside the HMG box(es) themselves. Correspondingly, their common characteristic is involvement in DNA binding, whereas they otherwise participate in diverse cellular processes and may operate by distinct biochemical mechanisms. The common feature of DNA binding is despite considerable sequence variability in the HMG box(es) themselves.

The HMG box is the sequence signature by which the class is recognized. ‘HMG box’ is also an accepted term for the corresponding folded protein domains. Here we will use the term ‘HMG box’ interchangeably with ‘HMG domain’ in the latter meaning, often referring to HMG domains when the intent is to emphasize their identity as physical subdomains of the protein.

### Canonical HMGB protein, HMGB1

**Figure 2:**
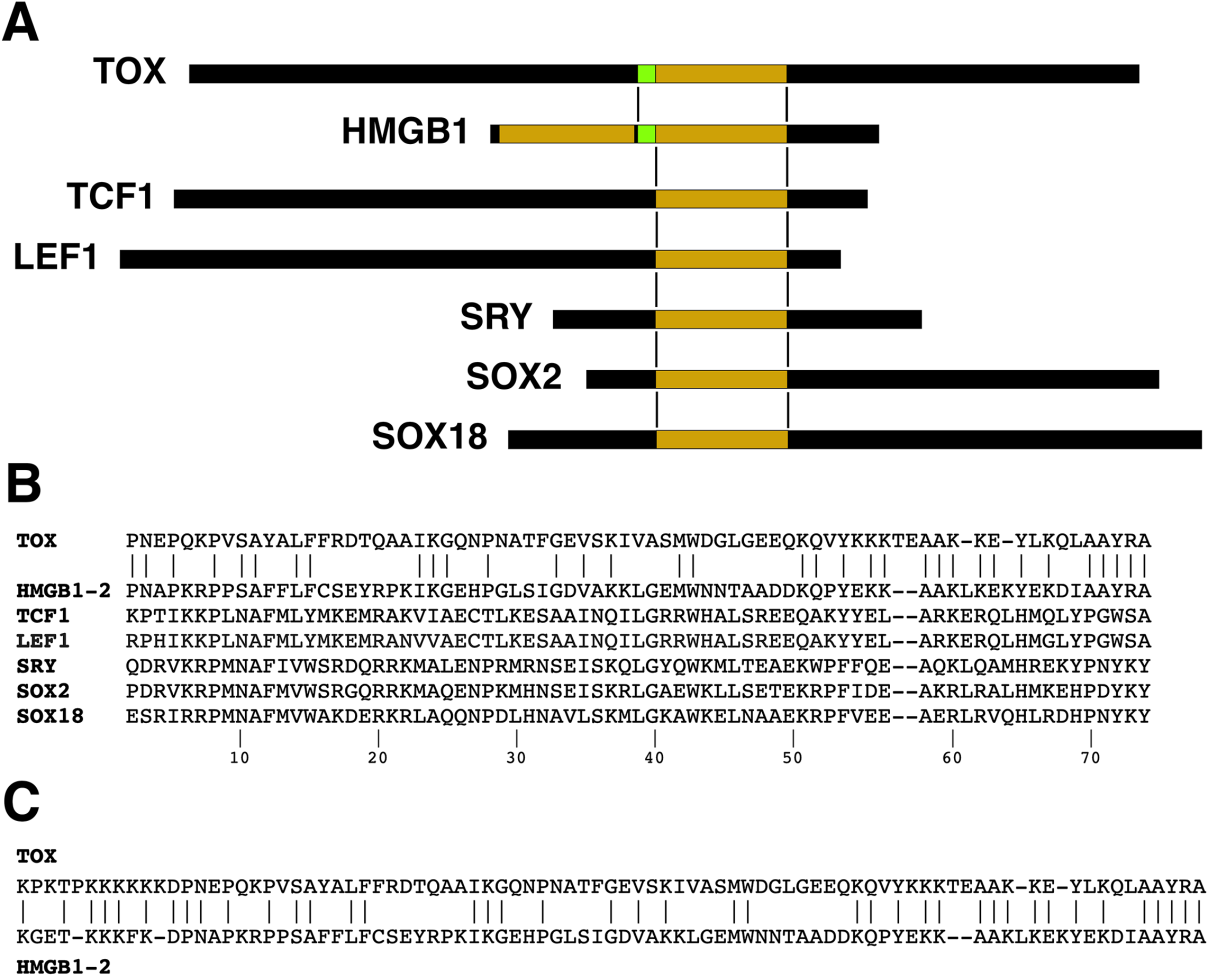
HMG domain alignments. **(A)** Schematic alignment of TOX with the second HMG domain of HMGB1 and a short segment of flanking sequence. Corresponding alignments of some other HMG-box proteins are also shown. The proteins lack substantial sequence homology with HMGB1 outside the HMG domain. **(B)** Sequence alignment of the HMG domains of human TOX, human HMGB1 (domain 2), human TCF1, human LEF1, human SRY, human SOX2, and human SOX 18. The numbering of positions within the HMG domain follows (32). Note that other ‘canonical’ numbering systems have been used in the literature. **(C)** The TOX-HMGB1(domain 2) sequence alignment extends a short distance into the N-terminal region flanking the HMG domain.

The defining member of the class is HMGB1, a relatively small protein of 215 residues in humans, comprising two HMG domains connected by a short linker region and exhibiting only modest pairwise identity; two nuclear localization sequences; and an acidic C-terminal tail (**Figure 2A**). We will focus on the way HMGB1 carries on an ancient role of similar proteins in DNA architecture and in processes that involve DNA flexibility and DNA rearrangements (DNA replication, DNA repair, transcription). Other established cellular functions of HMGB1 will not be addressed here.

### HMG domain alignments

The single HMG domain in human TOX can be aligned, by definition, with either HMG domain of HMGB1, but it has more sequence identity with the second HMG domain (**Figure 2B**). TOX exhibits 47% and 26% identity with HMGB1-(domain 2) and HMGB1-(domain 1), respectively, in the region covering the respective HMG boxes. For comparison, the alignments for human TCF1 (percent identity 23%), human LEF1 (22%), human SRY (26%), and human SOX2 (27%)— other proteins that possess a single HMG box and that will be discussed below— are shown in (**Figure 2B**). The TOX–HMGB1 homology extends to the linker region of HMGB1 on the N-terminal side of the second HMG domain, resulting in 48% identity over the region comprising TOX residues 246–330 (**Figure 2C**). Thus TOX adheres more closely to the HMGB1 sequence in its DNA-binding domain than these other familiar HMG-box transcription factors. The protein alignment also brings to the fore the fact that there is more to TOX than an HMG domain: Its N-terminal region of ~240 residues and C-terminal region of ~200 residues account for the bulk of the TOX protein, and exhibit no informative homology with other human or vertebrate proteins.

### Anopheles ‘TOX’

TOX homology with the predicted *Anopheles gambiae* protein now annotated as XP_061513506 was reported in an early description of TOX-family proteins (33). The conserved sequence matches the part of TOX that aligns with HMGB1 in the linker region and the second HMG box, and it extends five residues beyond the end of the TOX-HMGB1 alignment. Proteins with a similar region of alignment have since been predicted from some other insect genomes. These are frequently annotated as TOX, although homology to mammalian TOX proteins is lacking outside the indicated region, and there is currently no functional evidence linking this set of predicted proteins to TOX. The best tentative conclusion is that this constitutes an example of the reuse of a successful DNA-binding motif for different purposes during the evolution of some insect lineages and of vertebrates.

### Lessons applicable to vertebrate TOX

Recapitulating, the broad class of HMG box proteins does not form a protein family in the usual sense of having a common cellular function or functions. The HMG box has been reused often in evolution as a modular element incorporated into varied DNA-binding proteins and transcription factors. Therefore we can look to the HMG box of TOX for some understanding of its DNA binding characteristics, and we will do so here. The TOX HMG box in association with well conserved TOX N-terminal and C-terminal regions is a specialized adaptation of vertebrates. It will be necessary to look to these other regions of TOX for a more complete understanding of its biological and biochemical functions in vertebrate cells. We will touch only briefly on the questions that need to be addressed in this area.

## HMG box proteins stabilize bent DNA

### HMGB1-(domain 2)

**Figure 3:**
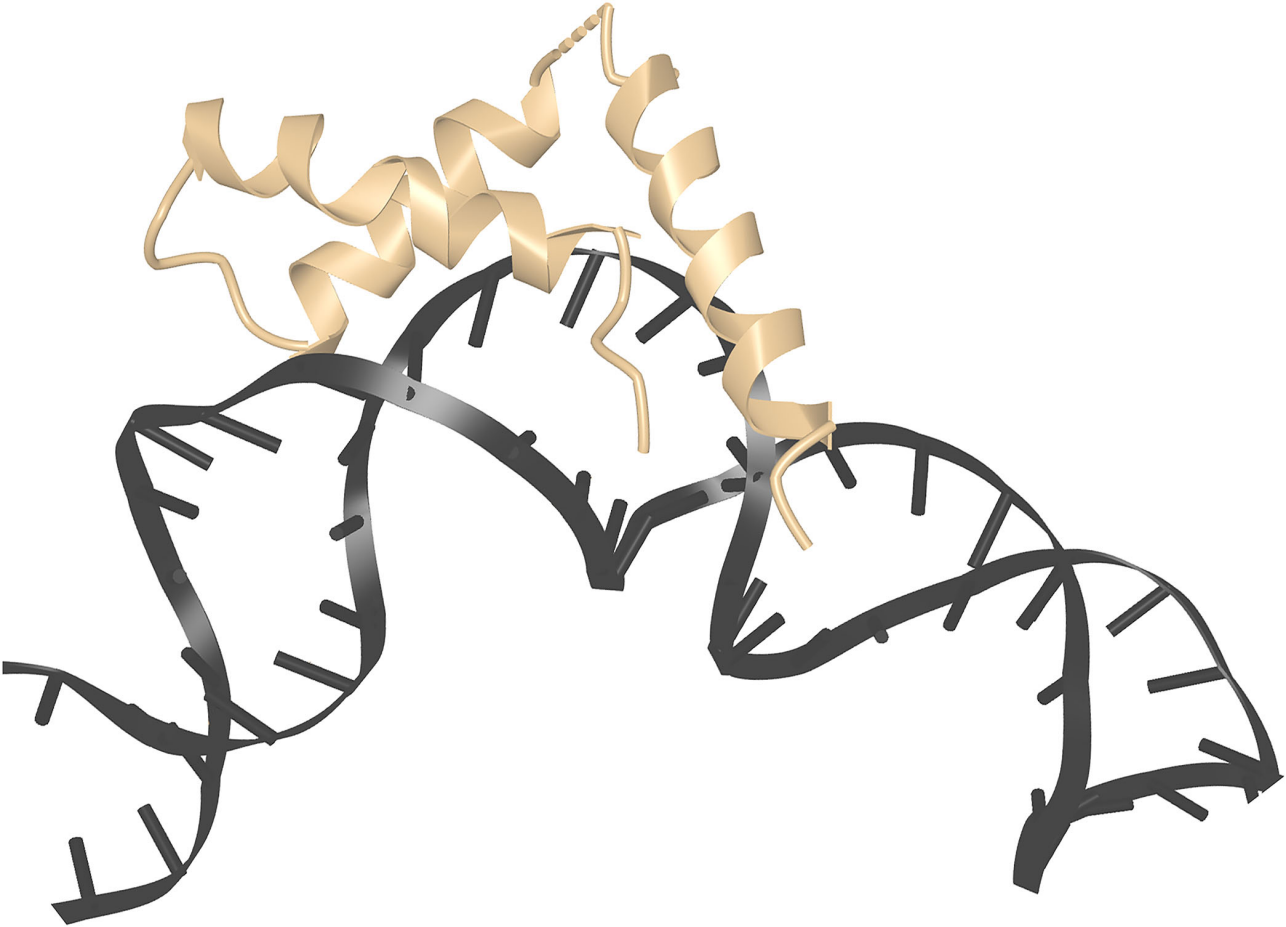
HMGB1 domain 2 stabilizing kinked DNA. Detail from PDB ID 6CIM (34). Structural data retrieved from MMDB (37) and viewed with Cn3D (38).

The structure of an HMGB1-(domain 2) complex with DNA has been determined as part of the RAG1/2 pre-reaction complex that forms on DNA prior to V(D)J recombination (34, 35). HMGB1 plays a supporting role in this process, since it is not essential for the coupled cleavage reaction catalyzed by recombinant RAG1/2 *in vitro*, but it can substantially increase the efficiency of the reaction (34, 36). HMGB1-(domain 2) contacts DNA in the minor groove and along the phosphate backbone. Its binding to DNA is associated with a localized distortion of DNA geometry, visible as widening of the minor groove and axial bending of the DNA helix by 90° (**Figure 3**). The local bending of DNA is supported indirectly by the linked HMGB1-(domain 1) and by interactions with RAG1/2 that specify the orientations of the distal parts of the recombining DNA segments, but only HMGB1-(domain 2) is in direct contact with DNA in the region of the bend.

### HMG-D, SRY, SOX-family proteins, LEF1

X-ray crystal structures or NMR structures of the HMG domains of a *Drosophila* homolog of HMGB1, HMG-D; SRY; the SRY-related proteins SOX2, SOX9, and SOX18; and LEF1 exhibit similar sharp axial bending of the DNA centered on the protein binding site (39–45). The estimated extent of bending varies with the precise protein construct and experimental conditions, and can be influenced by lattice packing in protein-DNA crystals. Values observed in SRY-DNA crystal structures ranged from 69°-84°, and values for published SOX-DNA crystal structures, from 54°-76° (41, 44); measurements on protein-DNA complexes in solution indicated similar or even more pronounced bending (LEF1, 117°; SRY, 54°; and SOX, 101°) (46). These cases represent diverse HMG box sequences, each comprising a single HMG domain, with less identity than TOX to the defining HMGB1-(domain 2) sequence. All these proteins, and others not cited here, can stabilize the bending of otherwise linear DNA in solution or in crystals of simple protein-DNA complexes, under conditions that ensure no other proteins are involved, even indirectly, in DNA bending. These findings do not, of course, prove that the HMG-box proteins act alone to bend DNA in cells and at their physiological protein concentrations.

### Minor groove distortion and energetics of binding

Sharp bending of DNA is required in the course of many cellular processes such as DNA replication, DNA repair, and mRNA transcription, yet it runs counter to certain ingrained expectations. The DNA double helix is typically portrayed in the literature as a rather rigid rod, with a stated persistence length ~50 nm, or ~150 bp. On the other hand, the fact that DNA wraps tightly around the histone octamer of a nucleosome is a reminder that DNA can bend locally under the right conditions (47). Local stiffness is not, in fact, an intrinsic property of the DNA helix itself. At least in AT-rich segments of the helix, it is imparted by the presence of highly ordered water molecules in the double-helix minor groove (48). The HMG domain is among several distinct protein adaptations that have evolved to bind in the minor groove, displace structured water, and enable the DNA flexibility needed for a variety of biological processes (49).

### Descriptions of DNA complexes

SRY serves as a textbook example of HMG-domain–DNA binding. The structural features underpinning specific binding are lucidly explained in (41). Overall, there is widening of the minor groove to accommodate the complexed protein, and narrowing of the adjacent major groove. Binding is supported by hydrogen bonds in the minor groove to DNA bases and by intraprotein hydrogen bonds that structure the protein-DNA interface, by van der Waals contacts in the minor groove, and by partial intercalation of an isoleucine side chain between DNA bases. Contacts on the DNA phosphate backbone attenuate the repulsion between negatively charged phosphates and thus support a narrowing of the major groove. The orientation of a few affected bases is twisted to accommodate the change from B-DNA geometry, but Watson-Crick base pairing is preserved. A high-resolution (1.75 Å) view of the closely related protein SOX18 bound to DNA shows a similar geometry and very similar hydrogen bonds (44). A slight difference is that arginine-20 of the SRY structure (termed arginine-18 in the SOX18 structure) retains the intraprotein hydrogen bonds that structure the interface, but has only van der Waals contact with DNA. The high resolution allows visualization of ordered water between that arginine side chain and DNA. Klaus et al. (44) discuss subtle rearrangements of protein-DNA contacts in other SOX-family crystal structures that accommodate variations in the sequence of the core DNA binding site. Other HMG domain-DNA structures display a similar or lower number of hydrogen bonds to DNA, and sometimes more reliance on van der Waals contacts, even to potential hydrogen-bond acceptors (39, 50). In some cases, binding is supported by intercalation of protein side chains into DNA at a second neighboring dinucleotide site (39, 51).

### DNA bending is confirmed by physical measurements

DNA bending has been confirmed independently in many cases by physical measurements on HMG domain-DNA complexes in solution. The simplest approach conceptually has been to make FRET measurements on oligonucleotides with a centrally located binding site and FRET donor and acceptor fluorophores placed at opposite ends of the DNA. Distances are estimated for unbound linear DNA and protein-bound bent DNA, and are consistent with the structures. ‘Permutation gel electrophoresis’ has also been informative. DNA fragments complexed with an HMG-domain protein that induces a centrally located kink or bend migrate more slowly on an agarose gel than protein-bound linear DNA of the same length. As the HMG-domain binding site is moved from the center toward either end of the oligonucleotide, the mass of the protein-DNA complex is unchanged, but the DNA geometry changes, and electrophoretic migration reverts gradually to the pattern for the linear oligonucleotide. Migration of oligonucleotide standards with a known degree of bending can be compared to determine bend angles.

### TOX in this context

**Figure 4:**
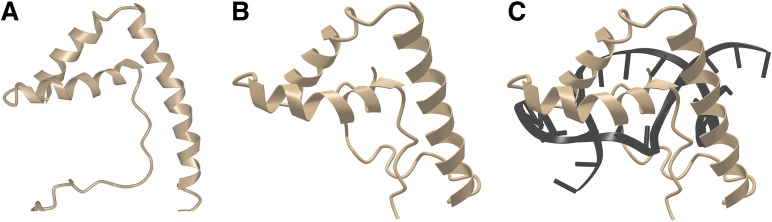
TOX closely resembles LEF1 in three-dimensional structure. **(A)** NMR structure of mouse TOX. From PDB ID 2CO9 (52). Structural data retrieved from MMDB (37) and viewed with Cn3D (38). **(B)** NMR structure of mouse LEF1, from PDB ID 2LEF (45). LEF1 was complexed with target DNA, but DNA is omitted from this view for direct comparison with the TOX structure in **Figure 3A**, Structural data retrieved from MMDB (37) and viewed with Cn3D (38). **(C)** NMR structure of mouse LEF1 in complex with DNA, from PDB ID 2LEF (45). Structural data retrieved from MMDB (37) and viewed with Cn3D (38).

An NMR structure of mouse TOX in the absence of DNA is available in the Protein Data Bank (52), although the structure has never been published in a journal article. TOX has the same fold as other HMG box proteins discussed above (**Figures 4A–C**). Although it has been reported that the isolated TOX HMG domain does not bend DNA when it forms a complex with an unspecified oligonucleotide (53), this negative finding could indicate merely that DNA bending requires sequences outside the TOX HMG domain itself, as has been found with some other HMG box proteins, or it could mean that the oligonucleotide used was not a physiological target that fully engaged the TOX HMG domain. The question bears future investigation with authentic TOX sites. However— taking into account the high sequence conservation relative to HMGB1 domain 2, the conserved protein fold, and the consistent finding that HMG domains bind to bent DNA— the strong expectation is that TOX will bend DNA or will stabilize bent DNA when bound to its physiological target sites.

## Recognition of target DNA sites

### ‘Sequence-specific’ versus ‘structure-specific’ HMG domains

HMG-domain proteins are commonly classified either as ‘sequence-specific’, that is, showing preference for binding a recognizable DNA consensus sequence; or as ‘structure-specific’, that is, having no experimentally recognizable DNA consensus sequence. The latter proteins are considered to select sites based mainly on the ability of DNA to assume a preferred kinked or bent geometry. This is an artificial distinction to an extent, first, because the consensus DNA sequences for the ‘sequence-specific’ factors SRY, SOX2, LEF1, and TCF1 center on AT-rich DNA that is intrinsically amenable to bending; and, second, because in binding the ‘sequence-specific’ factors do bend DNA. Conversely, ‘structure-specific’ factors have been shown to bind in certain cases at defined sites in DNA, rather than randomly, as exemplified in the RAG1/2 pre-reaction complex (34), or in the footprint of HMG-D on a DNA fragment from the *Drosophila ftz* locus upstream regulatory element (54), or in the footprints of the RNA polymerase I transcription factor UBF and the mitochondrial transcription factor TFAM on their target DNAs (55–57). It is therefore important that the frequent shorthand description of the structure-specific subset as ‘non-sequence-specific’ should not be read as conveying that the proteins are indifferent to DNA sequence.

Current practice is to assign these classifications based on the amino-acid residues present at a few telltale positions in the HMG domain (50), rather than from an experimental determination of DNA binding sites. Cases that have been closely examined indicate that there are subtle structural differences in the protein-DNA contacts of structure-specific *versus* sequence-specific proteins. The structure-specific group shows less reliance on direct hydrogen bonds to DNA bases, more reliance on water-mediated hydrogen bonds and van der Waals contacts, and a ‘looser’ protein-DNA interface (39). These structural differences come with corresponding differences in the thermodynamics of protein-DNA binding that are consistent with the looser interface (48). It is open to interpretation whether the two modes of protein-DNA interaction serve as two ways of achieving the same outcome of DNA minor-groove binding, as alternatives allowing a graded relaxation of DNA site specificity, or as a combination of those possibilities.

### Highly abundant HMG-domain proteins

A grouping that is arguably more relevant to the cellular functions of HMG-domain proteins is according to their expression level. Mammalian HMGB1 and HMGB2 stand out in this regard. They are highly abundant proteins, with several million copies of HMGB1 per cell (58, 59) (**Table 1**), and are members of the structure-specific class. Evolution may have favored their ability to bind a range of linear DNA sequences and their availability at high protein levels in the cell nucleus because these features contribute to the efficiency of numerous processes that call for transient kinking of DNA. Examples are support for Ig locus recombination by the RAG1/2 complex, cited above, and action in concert with DNA topoisomerase to maintain the speed of transcription in long gene loci; as well as general cellular processes like DNA replication, DNA repair, and chromatin reorganization. The high abundance of HMGB1 and its rapid exchange at most binding sites in the cell nucleus (63) will help to ensure its ready availability at sites where it is needed.

**Table 1:** Protein abundances from PaxDb.

Human CD8^+^ T cells (60–62)	
HMGB1	5638 ppm
HMGB2	2071
LEF1	47.2
TCF1 (*TCF7*)	115
TOX	22.3
RUNX3 *	157
NFκB p65 (*RELA*) *	139
NFAT1 (*NFATC2*) *	148
*Drosophila* (61, 62)	
HMG-D	907 ppm
Yeast (61, 62)	
NHP6A	320 ppm
NHP6B	172
GCN4 *	10.6
GAL4 *	17.0
PHO4 *	8.97

*Binds in the major groove of DNA.

Values retrieved from PaxDb: Protein Abundance Database (https://pax-db.org) 31 December 2024. Ppm values are estimated representation of the indicated protein as a fraction of all protein molecules in the sample. Gene symbols are noted where they do not directly mirror the protein nomenclature. The database entry for human CD8^+^ T cells is labelled ‘Cell line, Cd8’, but the source publication states clearly that the sample consisted of CD8^+^ T cells isolated from human peripheral blood.

HMG-domain proteins with similar high-level expression are found across species, typified by HMG-D in *Drosophila* and NHP6A and NHP6B in yeast. NHP6A is present at ~60,000 copies per yeast cell (64), which translates to a concentration in the yeast nucleus comparable to that of mammalian HMGB1 in the larger mammalian cell nucleus. The independent mass-spectrometric estimates of protein abundance in **Table 1** are consistent with the values just cited, after taking into account the typical volumes of a yeast cell (40 μm^3^) and a human lymphocyte (200 μm^3^) and the expected total protein density (~3x10^6^ proteins per μm^3^) (65). The seemingly depressed ppm values for NHP6A and NHP6B in **Table 1** compared to values for HMGB1 and HMGB2 reflect primarily the rather low nucleus/cytoplasm volume of *S cerevisiae*— the nucleus is ~7% of the cell volume (66) compared to more than half the cell volume in a T cell— and the consequent high representation of cytoplasmic proteins.

HMG-D, NHP6A, and NHP6B have single HMG domains, not a pair of domains as in HMGB1, but are thought to perform physiological functions akin to those of HMGB1 and HMGB2. Like HMGB1, they lack the signature residues of sequence-specific HMG-box proteins. Delivery of NHP6A to its sites of action may be assisted by one-dimensional facilitated diffusion of NHP6A along DNA (67), which could allow efficient scanning of DNA for relevant binding sites.

### Less abundant HMG-domain proteins

Most HMG-box proteins contrast with HMGB1 in having relatively low abundance in the cell. For example, LEF1, TCF1, and TOX are found at 50-fold to 250-fold lower protein levels than HMGB1 in human CD8^+^ T cells (**Table 1**). These levels are comparable to the levels of major-groove-binding transcription factors (**Table 1**). Thus, most human HMG-box transcription factors share with the familiar major-groove-binding factors a vexing quantitative problem of finding their physiologically relevant binding sites in a sea of chromatin.

A rough calculation illustrates the dimensions of the problem. LEF1 and TCF1 have core consensus DNA sequence (A/T)(A/T)CAAAG (complement CTTTG(A/T)(A/T) (68, 69), and SRY has the core consensus sequence ACAATG (complement CATTGT) (41). Calculating from the average GC content of 0.41 in human DNA, an exact match to each of these core sites would occur at 0.03-0.04% of positions in the human genome. Even allowing for some binding to inexactly matched core sequences, target sites are still sparsely distributed in chromatin. The less abundant HMG-box transcription factors cannot afford to tarry on nonspecific sites. Two strategies they might borrow from the repertoire of HMGB and NHP6A are relatively rapid release from interactions with non-target sites and a DNA-scanning mechanism.

Although the sequence-specific factors do find certain target sites reliably and drive predictable physiological outputs, as illustrated in some of the examples below, it should not be expected that all sites matching the consensus sequence are robustly occupied in cells. A recent breakdown of observed TCF1 binding sites in CD8^+^ T cells counted ~19,000 high-confidence TCF1 ChIP-seq peaks, with ~5000 of those lacking TCF1/LEF1 motifs and thought to recruit TCF1 through its interactions with other transcription factors (70). The core sequence specifies an asymmetric site, so it is necessary to consider locations on either strand of DNA, and the expected number of core TCF1 sites in the haploid human genome is ~0.04% of 6x10^9^ bp, or ~2.4 million sites. If attention is restricted to accessible chromatin— a few percent of the genome— the calculation indicates that there are, minimally, on the order of 50,000 accessible exact-match sites. There will be a considerably larger number of candidate binding sites after including sites with acceptable deviations from the consensus. The discrepancy can be accounted for to an extent by the presence of multiple copies of the core sequence in some peaks, but a visual scan through the genome confirms that isolated copies of the exact-match core sequence are not rare. The implication is that TCF1 and other sequence-specific HMG-box factors discriminate among potential binding sites based on DNA features beyond the core consensus sequence or cooperation with DNA-binding protein partners.

### Proposed TOX consensus site

TOX is assigned to the structure-specific group of HMG box proteins based on the signature distinguishing residues in its HMG domain (33). Nevertheless, Artegiani et al. stated that TOX binds preferentially to a GC-rich motif in HEK293T cells, based on labelling DNA sites using a TOX-DNA adenine methyltransferase (Dam) fusion protein and bioinformatic analysis (71). DamID-seq has the strength that it labels regions adjoining sites of protein-DNA interaction in live cells, and the limitation that labelling is integrated over a period of many hours— in this case 48h after infection with TOX-Dam lentivirus— and so could include repeated transient interactions in addition to stable TOX-Dam–DNA interaction sites (72, 73). TOX-Dam labelling was substantially increased at promoters (71), where GC-rich segments are common, and hence an alternative interpretation is that the bioinformatic enrichment merely reflected the abundance of promoter-proximal sequences in the dataset. Crucially, no data were offered showing preferential binding of TOX to the proposed consensus site. The recombinant TOX HMG domain did not bind to the site *in vitro* (74).

### Experimentally mapped TOX sites

**Figure 5:**
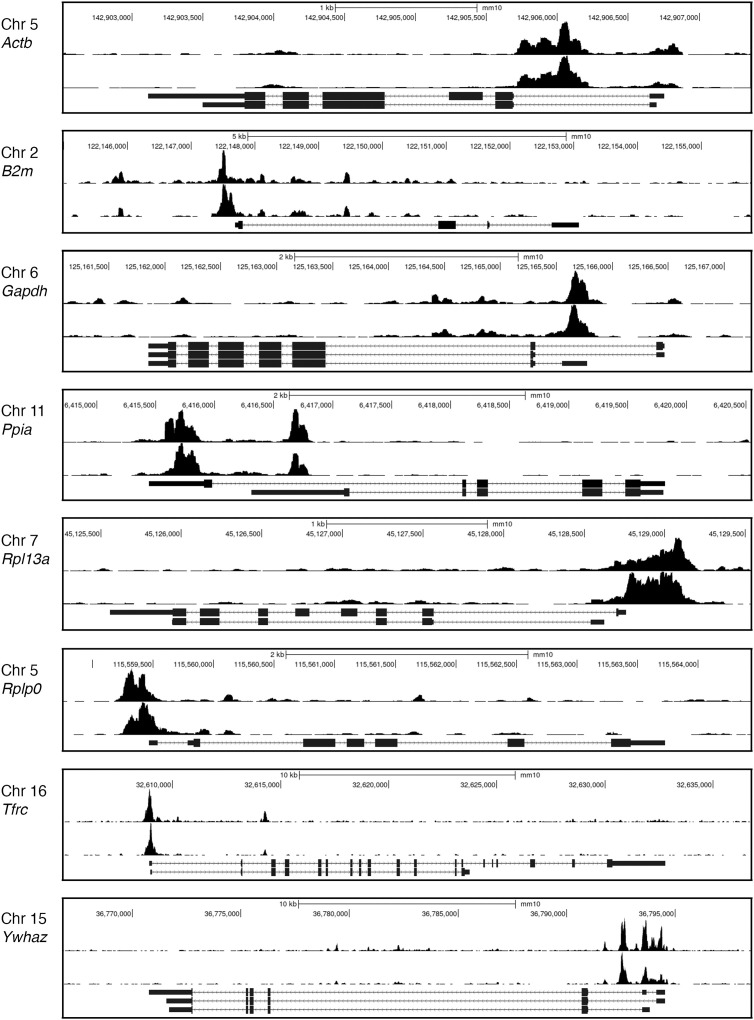
TOX CUT&RUN data. Representative Genome Browser views of the CUT&RUN signals for H3K27Ac (*upper track* in each panel) and TOX (*lower track*) at several housekeeping gene loci. The pronounced TOX CUT&RUN signals at promoters and enhancers of housekeeping genes in CD8^+^ TILs raise two questions— whether these signals faithfully reflect TOX positioning in living TILs and, if so, whether this distribution of TOX can be related to control of the exhaustion transcriptional program. Data from (77, GEO accession GSE175443). The examples shown are for PD1^high^TIM3^+^ TILs.

There have been further attempts to define TOX binding sites experimentally. One of these utilized CUT&RUN mapping (75, 76) to delineate TOX binding sites in CD8^+^ TILs that had infiltrated B16 melanomas (77). It is notable that the observed TOX signal often closely paralleled the H3K27Ac signal, even in housekeeping gene loci (**Figure 5**). TOX has been shown to coimmunoprecipitate with chromatin-binding and chromatin-remodeling proteins (10), so one plausible interpretation would be that TOX is prepositioned at promoters and enhancers to facilitate its normal cellular functions. A more skeptical view would be that TOX binding at its physiological DNA target sites is not stable during the prolonged CUT&RUN incubations of unfixed permeabilized cells, and that the observed binding at promoters and enhancers reflects a redistribution of TOX driven either by specific interactions with chromatin proteins or by nonspecific interactions with highly accessible chromatin.

Conventional TOX ChIP-seq identified candidate TOX peaks in CD8^+^ T cells from a CNS autoimmune disease model (28). A serious reservation in this case is that only single samples were analyzed for wildtype and for TOX^–/–^ T cells. There are many instances where an apparent TOX ChIP peak from wildtype cells is mirrored by a substantial but somewhat smaller peak from TOX^–/–^ cells, and, in the absence of experimental replicates, there is no way to assess whether the higher signal in wildtype cells is statistically significant.

### Implications for TOX

What can be said confidently of TOX is that it is a low-abundance HMG box protein and has the protein-sequence fingerprints of a structure-selective binder. What is expected is that its HMG domain by itself will exhibit moderately weak binding to DNA sites, but that regions outside the HMG domain might make a further contribution to TOX-DNA affinity. One such region is the basic stretch immediately preceding the HMG domain, which bears sequence homology to a comparable stretch in HMGB1 that is thought to increase the affinity of rat HMGB1 for DNA (46). Like the more abundant structure-specific HMG-box factors, the TOX HMG domain is likely to be adaptable in its DNA site preferences, by minor rearrangements of its side chain contacts in the minor groove. As noted already for SOX-family proteins, this adaptability is a feature shared with at least some sequence-specific factors, although it may come to the fore in structure-specific factors due to their less-tight contacts on DNA.

TOX, like other transcription factors, has the biological imperative to recognize useful sites. In light of the low protein copy number, the need to discriminate relevant sites from irrelevant sites becomes particularly acute. In some respects, this is not a completely different situation from that of the sequence-specific factors, which in practice also select a subset from among possible binding sites. However, with no guidance from DNA sequence, the delineation of relevant TOX binding sites will rely entirely on experimental data. TOX target sites in DNA are likely to vary with the cell type and developmental context, and, correspondingly, TOX protein partners may differ in different contexts.

## Examples of varied HMG-box protein functions

### HMGB1 with RAG1/2 in V(D)J recombination

HMGB1 has been visualized binding to DNA along with RAG1/2 recombinase in the run-up to V(D)J recombination (34, 35) (**Figure 3**). The recombination signal sequences ‘12-RSS’ and ‘23-RSS’ mark sites for recombination in V, D, and J gene segments. HMGB1 is seen bound to a loop comprising the 23-bp spacer of the 23-RSS in the X-ray crystal structure. Despite its lack of DNA sequence selectivity and the consequent theoretical possibility of its binding anywhere, HMGB1-(domain 2) here chooses a specific site, implementing the biological imperative that the longer stretch of DNA in the 23-RSS spacer has to bend to position it correctly in the RAG1/2-DNA complex. HMGB1-(domain 1) binds adjacent— itself stabilizing a local bend in DNA in conjunction with DNA contacts made by RAG1/2— and is required for efficient positioning of domain 2. The 23-RSS spacer sequences themselves are not strongly conserved in the 23-RSS segments of germline human and mouse immunoglobulin (Ig) loci, but Kim et al. note the frequent occurrence of bendable CA (complement TG) dinucleotides in a comparable position in these Ig gene segments, suggesting that evolution has provided an amenable local sequence appropriately positioned within this loop (34). This is a straightforward example of how the abundant HMG-box protein HMGB1 stabilizes a bent DNA conformation and enables efficient implementation of a cellular process.

### SOX2 in a transcription factor complex with OCT1

**Figure 6:**
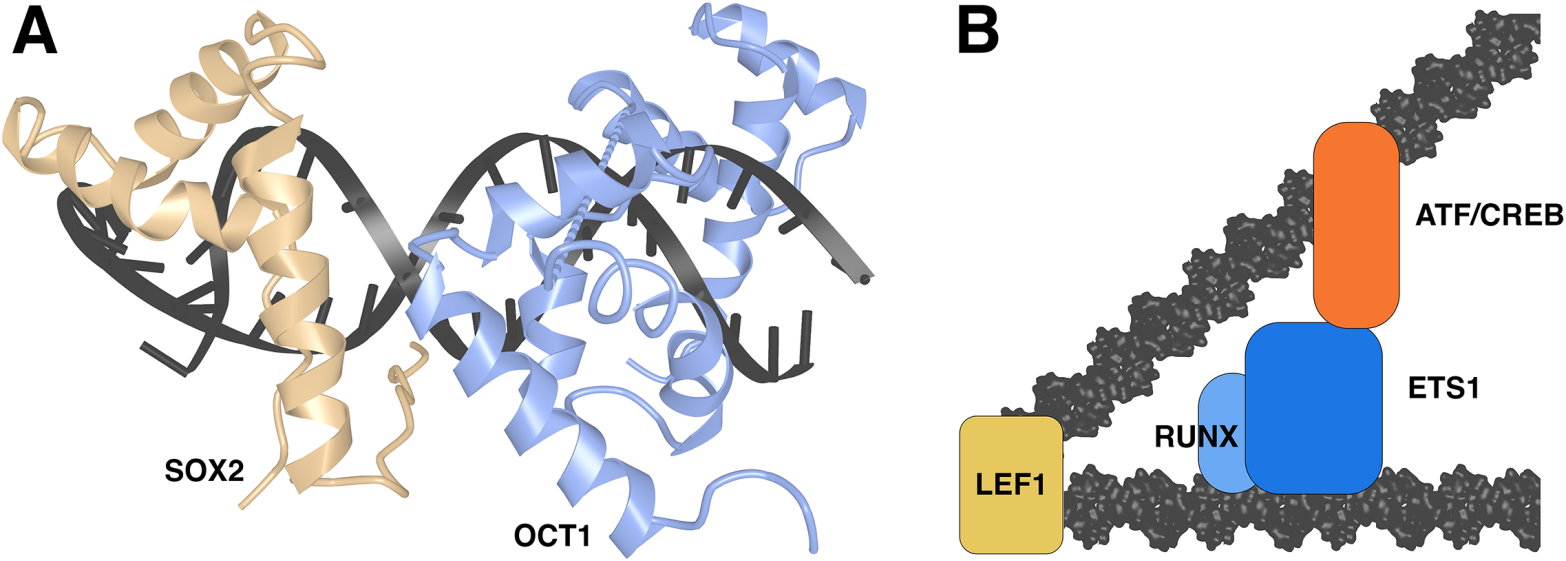
HMG-domain proteins in transcriptional complexes. **(A)** SOX2-OCT1 complex on DNA in the crystal structure PDB ID 1O4X (42). Structural data retrieved from MMDB (37) and viewed with Cn3D (38). **(B)** Schematic view of LEF1-ETS1-ATF/CREB complex. Based on (81, 82).

Low-abundance HMG box proteins can act as conventional site-specific transcription factors by forming complexes with protein partners. Drawing an example from the SOX family of transcription factors, whose roles in development have been reviewed in (78, 79), SOX-family proteins work together with partner proteins, including OCT-family proteins, to orchestrate diverse developmental processes (79). An X-ray crystal structure of the SOX2-OCT1 transcription factor complex on an *FGF4* enhancer and an NMR structure of the complex on a *Hoxb1* element (42, 80) (**Figure 6A**) portray the expected SOX-dependent bending of the DNA site, and protein-protein interfaces that are dictated by the relative geometries of bound SOX2 and OCT1 on the *FGF4* and *Hoxb1* DNAs. DNA bending is not directly involved in facilitating this protein-protein contact, but serves to narrow the minor groove beyond the SOX2 site, thus specifying the precise positioning of the C-terminal sequence flanking the SOX2 HMG domain. This placement of the C-terminal flanking sequence anchors the third α-helix of the HMG domain, and either the C-terminal flanking sequence or the third α-helix then presents as the binding surface for OCT1, depending on the geometry specified by the DNA site.

### LEF1 in a transcription factor complex

In another similar case, LEF1 acts at a defined TCRα minimal enhancer to promote mRNA expression (83). Here there is compelling biochemical evidence that LEF1 binding to the TCRα enhancer site acts in part by bringing together nearby binding sites for an Ets-1/RUNX1 complex and ATF/CREB, fostering assembly of a stable transcriptional complex (81, 82) (**Figure 6B**). Experimental evidence in these papers shows that DNA bending by LEF1 is a key to assembly of the full transcriptional complex. LEF1 also separately promotes transcription in this case via an action independent of DNA bending or CREB engagement in the complex (81, 82).

### SRY binding at a SOX9 enhancer

The sex-determining region Y protein, SRY, initiates the development of male gonads in mammals. Mechanistically, SRY drives induction of the SRY-box transcription factor 9 gene (*Sox9*) in cells of the genital ridges in mouse embryos, during a defined time window between embryonic days 10.5 and 11.5, provided that a threshold level of SRY is achieved in those cells (reviewed in 84, 85). Subsequently, SOX9— an SRY homolog, as its name indicates— takes over to drive its own continuing expression and the differentiation of cells expressing SOX9 as Sertoli cells, which then orchestrate the differentiation of other classes of testicular cells. Several enhancers in the mouse *Sox9* locus can bind SRY, but one key enhancer site for the early induction of *Sox9* has been defined, *Enh13*, and testicular development is abrogated when this enhancer is deleted (86, 87). SRY occupies sites in *Enh13* at day 11.5, as shown by ChIP-qPCR of a MYC-tagged SRY transgene (86). SRY acts as a conventional transcription factor in this case, and whether DNA bending is central to its action has not been determined. However, the example highlights the two key points that timing can be essential to the action of an HMG-box transcription factor and that a faithful biological output may require that a threshold level of the transcription factor is reached (reviewed in 84). Further underscoring the existence of threshold concentrations, SOX9 is expressed naturally, but at lower levels, in XX-genotype genital ridges that go on to develop into ovaries (86); and overexpression of SOX9 redirects XX genital ridges to develop into testes (88, 89).

### SOX2 as a pioneer factor

The role of SOX-family transcription factors in development has been mentioned above. One specific mechanism through which they direct cellular developmental choices is as pioneer factors that initiate the opening of nucleosome-bound chromatin. Readers are referred to (90, 91) for an overview of pioneer transcription factors, and to (92) for a thoughtful review of the biology and mechanisms of SOX-family transcription factors. The pioneering function of SOX2 has begun to be captured in cryo-EM structures of SOX2 or its close sibling SOX11, or of a SOX2-OCT4 complex, engaging a nucleosome (93, 94). The two cases differ in the location of SOX2 binding relative to the DNA entry and exit points on the nucleosome, raising the possibility that the detailed geometry of pioneer factor interactions may be specific to individual DNA sites, but a common feature is a local kink distorting the DNA to facilitate an initial partial release of DNA from its interaction with core histones.

### TCF1 in chromatin organization

TCF1 has a central role in the T cell lineage, working in conjunction with certain other transcription factors to enforce T cell-specific chromatin opening and T cell-specific gene expression in developing thymocytes (95, 96). TCF1 continues to ensure the normal functioning of mature T cells, not just by regulating the activity of promoters and enhancers in individual gene loci, but by specifying the three-dimensional organization of chromatin in the nucleus (70, 97, 98). Some mechanistic insight into this process has come from an investigation of how TCF1 ‘preprograms’ gene expression in CD8^+^ central memory T cells (T_CM_) (97). In those experiments, mice engineered to express Cre recombinase under the control of a *Gzmb* promoter rapidly deleted *Tcf7* upon activation of mature *Tcf7* ^FL/FL^ CD8^+^ T cells. The T cells mounted a normal effector response to a first LCMV Armstrong infection, even in the face of the concurrent deletion of *Tcf7*, and produced normal initial numbers of memory-precursor cells. However, the resulting TCF1-deficient memory cells exhibited a diminished response upon restimulation. The data suggested strongly that the recall response involved chromatin rearrangements within topologically associating domains that were ‘preprogrammed’ in resting memory cells by TCF1 occupancy of binding sites in gene promoters and distal regulatory regions, including specific TCF1 binding sites in glycolytic-pathway gene loci and in the *Id3* locus. Editing the latter sites, individually or in combinations, would provide a definitive test of whether they are essential to specify a TCF1-determined chromatin architecture in resting memory T cells and to prefigure the chromatin reorganization and gene expression during a recall response.

## The path to TOX mechanisms

This discussion holds several lessons for research on the cellular mechanisms of TOX in T cell exhaustion. Since TOX is not uniquely concerned with exhaustion, the first clear lesson is that it will be crucial to conduct the experiments during the initiation and progression of CD8^+^ T cell exhaustion or under conditions that mirror the progression to exhaustion. The correct conditions will ensure an amenable chromatin state and the presence of relevant protein partners. Mechanistic experiments will need to give attention to the timing and cellular concentration of TOX, in light of the perturbing effect of partial knockdown or haploinsufficiency of TOX in T cell exhaustion models (8, 10), and the quantitative rather than qualitative difference in TOX expression in CNS-infiltrating CTLs that cause disease compared to CTLs that do not cause disease (28, **Figure 2D**). A central task will be to catalog TOX binding sites in chromatin that contribute to the exhaustion program. Then, for individual sites in or adjacent to exhaustion gene loci, it will be a priority to define the TOX protein partners at those sites, keeping in mind that both the HMG domain and TOX regions beyond the HMG domain may participate in recruiting partner transcription factors or chromatin modulators. An ancillary question will be whether DNA kinking or bending is central to assembly of protein complexes at specific sites, or, conversely, how DNA bending might thwart binding of some transcription factors. The default expectation has been that TOX acts as a conventional transcription factor, enhancing or suppressing gene expression at individual gene loci. Current evidence is not decisive on whether TOX controls the overall transcriptional program of exhaustion by acting at many loci, or controls expression of a small number of key gene targets that then secondarily implement the program, or is concerned primarily with the expression of genes that promote survival and expansion of the exhausted T cell population. TOX might have roles other than as a conventional transcription factor, including an unexplored pioneer function or a role in orchestrating the overall three-dimensional architecture of chromatin as cells progress into the exhausted state. There is no shortage of work still to be done exploring TOX mechanisms.
